# Technologies for detecting and monitoring drivers' states: A systematic review

**DOI:** 10.1016/j.heliyon.2024.e39592

**Published:** 2024-10-18

**Authors:** Maged S. AL-Quraishi, Syed Saad Azhar Ali, Muhammad AL-Qurishi, Tong Boon Tang, Sami Elferik

**Affiliations:** a Interdisciplinary Research Center for Smart Mobility and Logistics (IRC-SML), King Fahd University of Petroleum Minerals (KFUPM), Dhahran, 31261, Saudi Arabia; b Research Center, Elm Company, Riyadh, 12382, Saudi Arabia; c Electrical and Electronic Engineering, Universiti Teknologi PETRONAS, Seri Iskandar, 32610, Perak, Malaysia; d Department of Aerospace Engineering, King Fahd University of Petroleum Minerals (KFUPM), Dhahran, 31261, Saudi Arabia; e Interdisciplinary Research Center for Aviation and Space Exploration, King Fahd University of Petroleum Minerals (KFUPM), Dhahran, 31261, Saudi Arabia

**Keywords:** Driver fatigue, Driver's state, Detection, Wearable sensors, Unwearable sensors, Deep neural network, Edge computing

## Abstract

Driver fatigue or drowsiness detection techniques can significantly enhance road safety measures and reduce traffic accidents. These approaches used different sensor technologies to acquire the human physiological and behavioral characteristics to investigate the driver's vigilance state. Although the driver's vigilance detection technique has attracted significant interest recently, few studies have been conducted to review it systematically. These studies provide a thorough overview of the most advanced driver vigilance detection method available today in terms of sensor technology for scholars and specialists. This research is geared towards achieving three main objectives. Firstly, it aims to systematically gather, evaluate, and synthesize information from previous research published between 2014 and May 2024 on driver's state and driving sensors and their implementation on detection algorithms. It aims to provide a thorough review of the present state of research on wearable and unwearable sensor technology for driver fatigue detection, focusing on reporting experimental results in this field. This information will be necessary for experts and scientists seeking to advance their knowledge in this field. Lastly, the research aims to identify gaps in knowledge that require further investigation and recommend future research directions to help address these gaps. This way, it will contribute to the advancement of the field and provide beneficial insights for future researchers.

## Highlights

• A reliable assessment of the driver's fatigue levels is vital for preventing accidents and ensuring road safety.
• Advanced approaches were used to assess the driver's state via wearable and Unwearable biosensors.
• Various sensing approaches implemented in the literature were investigated.
• Limitations and future directions were emphasized to enhance driving monitoring technology and improve road safety.

## Graphical abstract

**Figure:**
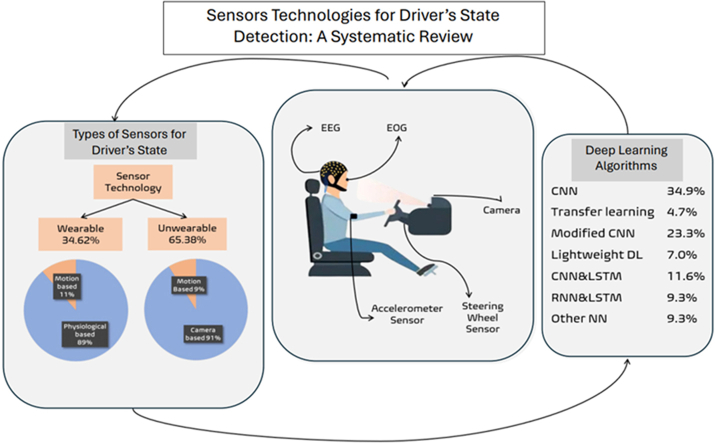

## 1 Introduction

In 2000, there were about 1.15 million road fatalities, while in 2018, there were about 1.35 million traffic deaths. Road traffic accidents rank as the eighth most common cause of death globally, contributing to around 2.37 % of the 56.9 million fatalities that take place each year [1]. Therefore, this road risk reflects the significance of detecting driver fatigue, which unavoidably resulted in an increased emphasis on this area of study. Aggressive driving, fatigue, health issues, ignorance of traffic safety, inconsistent and inefficient law enforcement, and sleepiness are the leading causes of these deaths [2,3]. The National Highway Traffic Safety Administration (NHTSA) revealed that 91,000 police-reported car incidents in 2017 included sleepy drivers [4]. Bus and heavy truck drivers should pay particular attention to this since they may have to work in tedious or dull situations during peak sleepiness [5,6], which can lead to accidents. Consequently, there is still a need for an intelligent system that can distinguish between fatigue and drowsiness with speed and accuracy [7]. In order to drive a car properly and react to situations that arise on the road, drivers require their entire attention when driving. It is a skill where the mind and body must constantly coordinate sophisticatedly. When circumstances arise that make it impossible for drivers to operate a vehicle safely, distraction results. According to Ref. [8], there are four types of driver distraction: visual (eyes off the road), manual (hands off the wheel), aural (listening to phone), and cognitive (thought of the task). Fatigue and drowsiness are among the fatal causes of driving safety dangers, along with speeding, drugs/alcohol, seat belt violations, and driver distraction.

Research indicates that driving while drowsy has similar effects as driving under the influence of alcohol [9,10]. Monitoring tired driving improves road safety and reduces fatalities and injuries. There have been reports of road accidents caused by fatigue and drowsiness. In general, driver's vigilance detection techniques may be categorized into four groups [[11], [12], [13]], based on the input characteristics: physiological signals [14], vehicle movement, subjective reporting [15], and facial expressions [16]. Real-time detection is less suitable for subjective reporting. The direct indicator of weariness is physiological signals. These techniques can be used with traditional machine learning, deep learning, or simple thresholding. Imaged or video-based techniques use computer vision and artificial intelligence to monitor the driver's face, eyes, mouth, or head nodding to predict fatigue or drowsiness.

On the other hand, the physiological-based approach uses biosignals such as Electroencephalography (EEG), electrooculogram (EOG), Electromyography (EMG) or skin conductance to measure the driver's brain activity, heart rate, muscle tension, or sweat level, respectively. These techniques require sensors or electrodes to be attached to the driver's body, and signal processing or machine learning methods are used to analyze the patterns and features of the biological signals. Additionally, vehicle movement analysis is based on vehicle-based indicators, such as steering wheel angle, lane deviation, braking time, or acceleration, to assess the driver's performance and behavior on the road. These techniques require sensors or cameras to be installed on the vehicle, and then statistical or machine-learning methods are used to detect anomalies or variations in vehicle movement. Furthermore, two or more detection approaches can be hybridized, such as image and biological signals, image and vehicle movement, or biological signals and vehicle movement, to improve the accuracy and robustness of the driver's fatigue detection [[17], [18], [19]]. Several studies have been conducted to detect the driver's vigilance to enhance road safety and reduce fatal road accidents.

Different sensor technologies were implemented to capture the driver's vigilance state. Recently, some researchers have surveyed different aspects of driver vigilance detection techniques. Several studies have reported on the progression of methodologies for detecting driver fatigue. A study by G. Sikander and S. Anwar [12], analyzes contemporary advancements in this field. The research scrutinizes various commercial systems designed to identify driver fatigue. It explores methods of fatigue classification based on features, dividing these into five categories: subjective reporting, biological indicators of the driver, the driver's physical manifestations, vehicle dynamics during operation, and a combination of these factors. Nevertheless, the investigation primarily concentrates on fatigue detection methodologies without considering the influence of various sensors on assessing the driver's alertness. Conversely, M. Khan and S. Lee [20] encapsulate research on distraction, fatigue detection, and the analysis of driving styles. Despite the broad scope, their review meticulously outlines the utilization of physiological sensors, including electroencephalograms (EEG) and electrocardiograms (ECG), for detecting driver distraction. They also cover the development and recognition of driving behaviors, and the technologies crafted to mitigate vehicular collisions.

This survey emphasizes the employment of physiological sensors for invasive driver monitoring, omitting a discussion on alternative, non-invasive sensor technologies for data acquisition. C. Zhang and A. Eskandarian [21], further narrow their research focus to using EEG sensors to monitor driver conditions, detailing the prevalent EEG system configurations for such studies alongside signal preprocessing, feature extraction, and classification methodologies. In a related study, H. V. Koay et al. [22], presented a synthesis of techniques for detecting driver distractions through machine learning, offering an exhaustive review of the diverse strategies employed. However, it was discovered that fewer efforts had been made to efficiently audit these studies as a way of providing analysts and specialists with an overview of the current sensors' technology in terms of wearable and unwearable settings, their potential applications in real-time vigilance detection, detection accuracy, durability in long-term detection, and different environmental conditions. Therefore, this study has three primary goals. Firstly, the aim is to systematically gather, condense, evaluate, and arrange data on the driver's state detection techniques from previous research published between 2014 and May 2024. Secondly, to comprehensively report on the current research results with a clear understanding of the current state of research regarding the use and benefits of sensor technology for detecting driver vigilance. Thirdly, it aims to identify all the data that requires a thorough investigation of its limitations and propose future research methods in driver's state detection techniques. The following research questions (RQs) have been proposed to achieve these objectives: (Q1) What types of wearable or unwearable sensors are used for the driver's vigilance? (Q2) What deep learning algorithms are used for driver's vigilance detection? (Q3) How are these sensor and deep learning algorithms implemented in real-time applications? (Q4) What are the positive and negative aspects of various sensor technologies? (Q5) What are this research area's challenges and future directions?

## 2 The review method

### 2.1 Search strategy

**Fig. 1:**
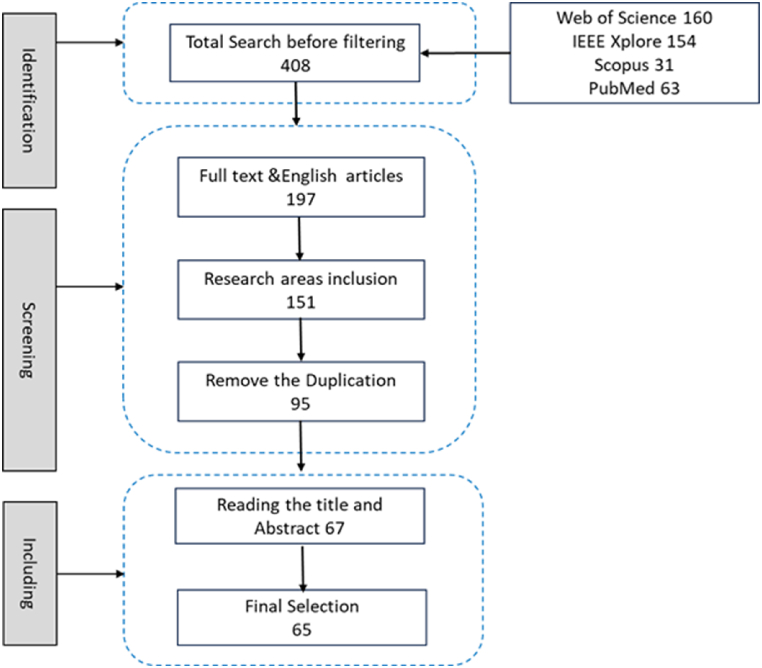
Search strategy flowchart.

We performed a thorough literature search on Web of Science, IEEE Xplore, Scopus, and PubMed, as shown in Fig. 1. The study only included full-text publications published in English, and the search encompassed items published between 2014 and May 2024. The combinations of search terms [Driver AND (Fatigue OR Mental fatigue OR physical fatigue OR drowsiness OR drowsy OR vigilance) AND (Detection OR Estimation OR Prediction OR monitoring) AND (Physiological OR EEG OR EOG OR ECG OR face OR eyes OR Behavioral) AND (Deep learning OR neural network) AND (real-time OR online)] were used. This study identified 408 articles after searching using the search keywords. Of this amount, 211 papers were filtered out by the criteria of full text, journal article and English text. Regarding research areas such as Engineering, computer science, neuroscience, healthcare, and transportation, 46 articles were excluded. The remaining 151 papers pass through the duplication removal process using Mendeley software. After the duplication procedure, 54 papers were removed. After reading the full text of the 95 remaining papers, a total of 30 papers were excluded, leaving 65 papers for this research.

### 2.2 Data extraction

General features were abstracted from the selected article, such as Data set type, Number of participants, type of detection techniques, Sensor used, measure and quantity and detection model utilized. Since this review focused on driver fatigue or drowsiness detection, some articles were excluded because they worked on driver behavior, such as work reported in Ref. [23]. After evaluating each research abstract separately, the authors concluded which studies might meet the requirements for inclusion. Full-text content was available for the articles that met the inclusion criteria. The categories for the articles included wearable and non-wearable sensors, detector-based techniques, and hybrid approaches for detecting or measuring the driver's condition.

## 3 Results

**Fig. 2:**
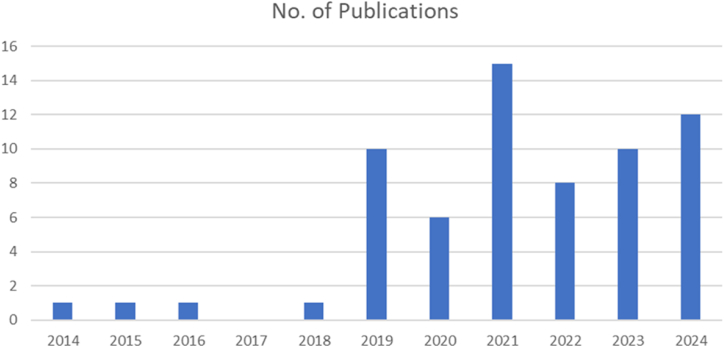
Temporal distribution of the selected publications in the Interval of 2014 to May-2024.

As indicated in Table 1, Table 2, Table 3, Table 4, 65 publications in the field of research on Driver's state detection approaches were finally selected and summarized as principal studies. In order to emphasize more how the Drivers' state detection has drawn more attention in recent years, Fig. 2 illustrates the temporal distribution of the selected publications from 2014 until May 2024. The trend in Fig. 2 shows an increase in the publications with time.

**Table 1:** Image based technique.

Ref	Year	Experimental Setup	Number of Subjects	Driving Duration	Type of Sensor	Physical Measurand	Detection Algorithm	Problem Solved	Limitations
[24]	2024		Public data set		Web Camera	Fatigue	CNN	The Cyber-Physical Systems are used to facilitate the real-time monitoring and analysis of the driving situation	It has a limited data set that will affect the model generality. The model must be validated with real-world challenges, such as lighting conditions.
[25]	2024	Driving simulator	Multiple drivers		Infrared cameras	Fatigue	Transfer learning with YOLOv8	The study proposed a fatigue-driving detection model that blends transfer learning methods with the YOLOv8n architecture.	The accuracy of fatigue identification can be impacted by individual variance because the model used in this work depends on factors like yawning and eye closure, which may not be relevant to all drivers.
[26]	2024		37 Subjects+ Public data set		Web Camera	Drowsiness	Deep Residual Networks (ResNet)	This study developed a deep learning architecture integrating residual and feature pyramid networks (FPN) to identify driver drowsiness.	Image quality, where the system's effectiveness relies on the quality of the input images. Additionally, the sensitivity of the camera position is essential.
[27]	2024		Public data set		Web Camera	Drowsiness	CNN and LSTM	The study combined IoT technology DL to develop an effective and unobtrusive drowsiness detection system. This integration allows real-time monitoring and notifications, which are critical for avoiding accidents.	Standard binarization algorithms struggle with dark skin tones, and limited variability in training data can affect their effectiveness. Testing conditions restricting rapid head movements may not accurately reflect real-world driving situations, impacting drowsiness detection.
[28]	2024		YAWDD dataset		Web Camera	Drowsiness	Deep Transfer Learning	The proposed method is intended to reduce false detections by employing ensemble learning and deep transfer learning models to ensure that only genuine drowsiness states are detected.	The accuracy of the DDD system depends on high-quality image processing. Factors like wearing sunglasses, changes in lighting, and camera-to-driver distance affect detection performance.
[29]	2024	Driving simulator	Public dataset		Web Camera	Drowsiness	lightweight convolutional neural networks	The suggested method employed lightweight CNN to ensure the system performed seamlessly on embedded devices such as Jetson Nano. This is essential for real-time applications.	The dataset has diverse photos; however, black-and-white images and varied lighting in the YALEB dataset can be challenging for lightweight models. The suggested network is lightweight but slightly more computationally complex than other networks.
[30]	2024	Driving simulator	37 subjects and two more online datasets		Web Camera	Drowsiness	two-branch multi-head attention architecture (TB-MHA)	The research presented a method for improving discrimination between various classes of samples (drowsy vs. awake) using a spatial filtering technique based on the Common Spatial Pattern (CSP) algorithm.	The technique detected fatigue mainly using facial features like landmarks and local areas. Still, its performance may be limited if facial visibility is compromised, such as when a driver wears sunglasses or has a partially concealed face.
[31]	2023	Actual driving	25 Subjects		RGB Camera	Drowsiness	Embedded system, edge computing, cloud computing modules	The study described an IoT-based automated approach for detecting Driver drowsiness This framework combined several components, such as an embedded platform, edge computing, and cloud computing, to give a solution for drowsiness detection and monitoring.	Reflections from drivers' spectacles can cause misclassifications and Reduced detection accuracy. Changing environmental conditions can also impact the system's performance, and there are potential data privacy risks with the cloud-hosted database for real-time monitoring.
[32]	2023	Driving simulator			Web Camera	Drowsiness	CNN model	The study proposed a method to detect driver fatigue by analyzing eye and mouth movements using a camera and a Convolutional Neural Network (CNN).	The current methods may fail at night or in poor conditions. Additionally, the driver's head posture can affect detection accuracy. The proposed model needs more real-world testing to assess the system's efficacy.
[33]	2023	Simulation	Multiple public data sets		Web Camera	Drowsiness	CNN model	This study examined the difficulty of achieving high precision on low-cost embedded devices like the Nvidia Jetson Nano. This makes the technology more practical for car use, improving safety.	The suggested method mainly detected signs of fatigue via the eyes and face. It did not look at other signs of fatigue, like posture and overall behavior, which could give a complete picture of a driver's condition.
[34]	2023	Actual driving	30 subjects		Web Camera	Yawning detection, fatigue detection	3D deep learning network and Bi-directional long short-term memory (LSTM)	This research addresses a few critical issues in yawning detection, particularly driver weariness, such as head posture variability, redundant frames, and lighting differences.	The detection accuracy depends on video data quality, including resolution, frame rate, and camera angle. It may also disregard minor yawning gestures between frames, resulting in false negatives.
[35]	2023	Driving simulator		1 h	Event camera	Yawning detection	lightweight deep learning models	It introduced event cameras, a neuromorphic sensing technology, for monitoring and verifying seatbelt fastening and unfastening. This approach used the unique capabilities of event cameras to give real-time analysis of dynamic behaviors that regular RGB or NIR cameras may not adequately capture.	The deployment of the proposed models within the limits of embedded hardware commonly seen in DMS is recognized as a difficulty. This constraint could impact the practical application of the system in actual automobiles, where computer resources may be limited.
[36]	2023	Simulator			Web Camera	Drowsiness And Fatigue	Threshold and DL based	The critical issue is the requirement for real-time monitoring of driver drowsiness. The suggested method provides instant feedback based on the driver's eye state, which is critical for prompt interventions to prevent accidents caused by drowsiness.	Drivers wearing sunglasses impair the model's effectiveness by preventing visual landmark identification and eye blink measurement, which are crucial for detecting fatigue. Facial landmark detection might also mislead the tracking driver if other objects block the face.
[37]	2022	Simulator	Multiple public data sets		Web Camera	Drowsiness	Various CNN models	The study introduced a real-time driver disturbance monitoring approach based on Convolutional Neural Networks (CNN). This technology is designed to assess driver drowsiness and fatigue, two crucial elements in road safety.	Implementing the proposed system in real-time applications may provide hurdles, notably in processing speed and computational requirements. The demand for high-performance hardware may limit technology accessibility in regular vehicles.
[38]	2022	Simulator	5 subjects		Camera	Fatigue	Machine learning and Resnet-50 models	This research aimed to develop a system for detecting driver fatigue by evaluating changes in facial features, particularly the eyes and mouth, in real-time.	The results showed that although some classifiers worked well, others did not. This variability indicates the system's performance may be inconsistent across different subjects or conditions.
[39]	2022	Actual driving	13 Subjects	1.5 h	Web Camera	Sleepiness	generic deep feature extraction module	The authors proposed personalized methods for identifying fatigue in drivers. They demonstrated the necessity of personalizing, as different people show signs of fatigue.	The authors acknowledged the limitations of their dataset, which was utilized to design and test the sleepiness detection system. They proposed that future research include independent datasets to validate these findings further.
[40]	2022		public data set		Web Camera	Drowsiness	two-stream spatial– temporal graph convolutional network (2s-STGCN)	This paper introduces the twin-stream spatial-temporal graph convolutional network (2s-STGCN). Many current driver drowsiness detection technologies face difficulties differentiating between various driving states, such as chatting, yawning, and blinking. This strategy sets out to rectify that.	The performance of the 2s-STGCN may deteriorate in challenging driving scenarios. Variations in lighting, shadows, and occlusions can all substantially impact the feature extraction process, potentially leading to misclassifications of driver states.
[41]	2022		public data set		Web Camera	Fatigue	multigranularity Deep Convolutional Model	The research proposed the Multi-Granularity Network (MEN), which used cues from partial face regions (such as the eyes, mouth, and glabella) to improve feature representation. This method addressed pose variability and enhanced the resilience of the feature extraction process.	The research acknowledged that the RF-DCM model cannot capture temporal information adequately. This limitation may impair the model's capacity to assess long-term relationships in time series data, critical for effectively diagnosing fatigue states over lengthy durations.
[42]	2022		public data set		Web Camera	Fatigue	(CNN)	This paper presented a systematic three-phase detection approach incorporating facial feature extraction, the Viola-Jones algorithm for identifying face traits, skin segmentation to ensure lighting invariance, and template matching for yawning and eye tracking.	This study revealed that drivers who wear glasses exhibit less precision in identifying facial characteristics and eye movements. The system's precision was inferior under low light conditions compared to daylight.
[43]	2021		synthetic event-based dataset		Neuromorphic vision sensors	Fatigue and drowsiness detection	(CNN)	The authors aimed to improve eye blink identification and analysis by developing a synthetic event-based dataset with accurate bounding box annotations, taking advantage of event cameras' high temporal resolution to improve overall driver safety and monitoring capabilities.	One crucial issue is the limited availability of event-based data, making it difficult to apply machine learning algorithms to event cameras successfully. The lack of data makes conducting thorough and statistically meaningful testing of the proposed approaches difficult.
[44]	2021	Simulator	8 Subjects		Web Camera	Fatigue	Detection fuzzy neural network	This study used an enhanced face identification approach to identify the driver's face in images collected by a CCD camera. It then used an ensemble of regression trees to identify face feature points, specifically the eyes and mouth.	The variation in recognition rates depending on head movement is one major limitation. Another significant issue with real-time face recognition systems is how varying lighting conditions or partially hidden facial features affect the system's effectiveness.
[45]	2021		Multiple Public data sets		Web Camera	Fatigue	CNN	In order to improve real-time performance and detection accuracy on edge computing devices, this research suggested a driver fatigue detection system that uses an optimized face alignment algorithm and a convolutional neural network.	The system's facial alignment performance may degrade under extreme conditions, such as when the driver is wearing sunglasses or in low-light situations. This can diminish detection accuracy, which is critical for real-world applications.
[46]	2021	Actual driving	10 Subjects	1 h	CCD Camera	Fatigue	Lightweight neural network model	In the context of vehicle-mounted embedded sensors with constrained memory and processing power, this paper attempted to address the problem of real-time driver fatigue detection using deep learning techniques on face video data.	Although the study claims to reach a detection speed of 27 FPS, more is needed in some real-time applications, particularly high-speed driving scenarios. Variations in the processing power of different vehicle-mounted embedded devices may alter the model's performance.
[47]	2021	Simulator	Public Data Set		HD camera	Yawning and Fatigue Detection	3D convolutional and BiLSTM networks	This paper presents a novel keyframe selection technique that reduces computational expenses and eliminates unnecessary frames from frame sequences. Achieving rapid detection of the most relevant frames is crucial for real-time processing.	The effect of low image quality on detection accuracy is one significant limitation. Additionally, significant camera vibrations might result in missing or false detections since face features are not always captured accurately.
[48]	2020	Simulator	Public data set		Web Camera	Drowsiness	A depth wise separable 3D convolutions	The study described a real-time method for detecting driver drowsiness using mobile platforms. The research emphasizes the usefulness of depth-wise separable 3D convolutions, which enable spatial and temporal data integration.	The method demanded significant processing capacity, notably for inference of 10-frame sequences. This high demand may limit the viability of real-time applications on lower-powered mobile devices. Because of these computational limits, the report suggests that other ways may be faster right now.
[49]	2020	Simulator	Public data set		Web Camera	Fatigue	Multi-task CNN model	The study suggested a Multi-task ConNN model using eye and mouth characteristics to measure driver fatigue. The model used the percentage of eye closure (PERCLOS) and the frequency of yawning as essential markers to determine the fatigue level of drivers.	The study used a constant frequency range and a set number of frames for analysis. This rigidity may limit the model's flexibility to diverse driving situations or surroundings, possibly affecting its real-world performance.
[50]	2020	Simulator	Public data set		Web Camera	Fatigue	a deep cascaded convolutional neural network (DCCNN	The research developed a Real-time and Robust Detection System (R2DS), to improve the accuracy, speed, and robustness of fatigue detection. This framework contains three main modules: facial feature extraction, ocular area extraction, and fatigue detection.	While the DCCNN is intended to increase detection accuracy, the study recognizes that methods based on artificial neural networks (ANN) frequently exhibit inadequate real-time performance due to their complicated structures and the requirement for considerable training data.
[51]	2019	Simulator	Public data sets		surveillance digital camera	Fatigue and Drowsiness	hybrid of CNN and (LSTM)	The research proposed the Eye Feature Vector (EFV) and Mouth Feature Vector (MFV) as evaluation parameters for determining the driver's eye and mouth states. These vectors are critical for estimating fatigue levels based on visual cues like eye closure and yawning.	The model detected fatigue primarily through facial indicators such as eye and mouth states. This concentration may neglect other signs of fatigue, such as physiological signals or behavioral patterns, which could provide a more comprehensive knowledge of a driver's status.
[52]	2019	Simulator	Public data sets		Web Camera	Drowsiness	A deep cascaded Convolutional neural network	The research described a condition-adaptive representation learning method for detecting driver drowsiness under various driving scenarios, including varied times of day and changes in the driver's appearance.	The proposed model requires a lot of labeled training data to cover various driver circumstances and scenarios appropriately. This can be a restriction when gathering such extensive data is unfeasible.
[53]	2019	Simulator	Public data sets		Web Camera	Drowsiness	convolutional control gate based recurrent neural network (ConvCGRNN)	The article developed a deep neural network (DNN) that can identify driver drowsiness in real time using video data. The network used CNN and ConvCGRNN, as well as a voting layer, to assess temporal relationships in facial data taken from videos.	The model's performance varies depending on the scenario, such as when drivers wear sunglasses or spectacles, which can obscure face features. Furthermore, the system depended substantially on consistent and precise facial tracking.
[54]	2019	Simulator	Public data sets		Web Camera	Drowsiness	Condition adaptive representation learning framework based on CNN	This approach combined four major models: spatiotemporal representation learning, scene condition understanding, feature fusion, and sleepiness detection. This system is intended to handle varied driving conditions in an adaptable manner and has been validated using the NTHU drowsy driver detection video dataset.	A significant limitation of this work is that the framework requires a large amount of labeled training data to handle different driving situations and scenarios adequately. Collecting such extensive data can be challenging.
[55]	2019	Simulator	10 subjects		Web Camera	Fatigue	Multiple Convolutional Neural Networks (CNN)-KCF (MC-KCF)	The system used a detection algorithm based on 68 critical facial locations, allowing for exact identification of facial regions crucial to drowsiness detection. This improved the system's accuracy in detecting indicators of weariness.	Drivers' heights can influence the location of their faces in the camera frame, leading to detection accuracy discrepancies. This diversity can make it challenging to ensure consistent performance for a broad group of users.
[56]	2018	Simulator	9 Subjects		Web Camera	Fatigue and drowsiness detection	Transfer learning classifier based on fast wavelet transforms and separator wavelet networks	The first essential contribution is the development of an eyes classifier that uses two transfer learning classifier designs. The second contribution is designing a fuzzy logic decision assistance system that divides driver vigilance into five categories.	Although the system is intended for real-time processing, the efficiency of the methods utilized (such as the Viola and Jones algorithm) can vary depending on the computational resources available. In resource-constrained contexts, this may impact system performance and dependability.

**Table 2:** Physiological based detection.

Ref	Year	Experimental Setup	Number of Subjects	Driving Duration	Type of Sensor	Physical Measurand	Detection Algorithm	Problem Solved	Limitations
[57]	2024		Public data set, SEEDVIG dataset		EEG and EOG	Vigilance	CNN with transformer	Contrastive learning enhanced the correlation between high-level EEG and EOG profiles before fusion. This approach confirmed that the features offered reliable information for multimodal fusion, thereby enhancing vigilance estimation.	Understanding the representation learning methodology employed with time series data is complex. The absence of explicit mappings between data features and prominent representation variables might complicate the comprehension and reliability of the model's predictions. Revise these learning mechanisms to enhance their interpretability in future research.
[58]	2024	Simulator	Two Public FD-EEGDS1 and FD-EEGDS2		EEG	Fatigue	FD-LiteNet	The study used neural architecture search (NAS) to automatically identify lightweight and high-performing CNN models for EEG-based driving fatigue detection. This strategy helps deploy resource-constrained devices in intelligent vehicles.	The study strictly searched for hyperparameters, ignoring architectural building elements like layer number and type. This limitation may prevent the discovery of better architectures that could boost performance. EEG signals are complicated due to individual traits, environmental factors, and artifacts.
[59]	2024	Simulator	27 healthy subjects	90 min	EEG	Drowsiness	Graph neural networks (GNNs) with GRU	This study proposes a connectivity-aware graph neural network (CAGNN) that uses self-attention to construct task-relevant connectivity networks for predicting drowsiness using EEG data.	Although the model had reduced intersubject variance than CNN-based models, subject variance remained substantial. Furthermore, the transformer design used in the model has been shown to cause overfitting when applied to smaller EEG datasets, which may jeopardize the model's generalizability.
[60]	2024	Simulator	Two online data sets		EEG and EOG	Fatigue	Generative ARMFCNLSTM (GARMFCNLSTM)	The authors presented two models, ARMFCN-LSTM and GARMFCN-LSTM. These models use adaptive multiscale temporal convolutions to extract multiscale representations automatically. GARMFCN-LSTM added a Wasserstein GAN with gradient penalty (WGAN-GP) to the ARMFCN-LSTM framework, which improved performance by managing data shortages and class imbalances.	Despite the excellent performance of the presented models, they are still dependent on the quality and quantity of training data. The dilemma of class imbalance and data insufficiency means that models may not perform best in circumstances with varying data distributions. Furthermore, the computational efficiency of the models may be an issue, especially for real-time applications.
[61]	2023	Simulation and actual driving	20 healthy subjects	1 h	EEG and ECG	Fatigue	Product fuzzy convolutional network (PFCN)	The study addressed the difficulty of accurately identifying driving fatigue by combining EEG and ECG readings, even in noisy environments. The author presented a Product Fuzzy Convolutional Network (PFCN) comprising three subnetworks to improve the robustness and accuracy of tiredness detection.	One notable drawback is how individual differences affect the model's detection accuracy. Furthermore, while the PFCN is more resilient and accurate than other models, the authors acknowledged that additional improvements are required to handle this variability adequately.
[62]	2023	Actual driving	12 subjects	3hrs	EEG	Fatigue	Wavelet Scattering Transform	The authors developed a classification method that uses a wavelet scattering network (WSN) to assess EEG signals obtained from drivers. The study showed that the WSN can extract significant elements from EEG data.	While the paper used the WSN algorithm to simplify the hyperparameter setting procedure, typical deep learning methods such as CNN and LSTM require substantial adjusting of several hyperparameters. This complexity can be a hurdle to practical implementation; however, the WSN technique mitigates some of these issues.
[63]	2021		Public data set		Single channel EEG	Drowsiness	CNN	The research proposed a dual one-dimensional CNN architecture for detecting fatigue using single-channel EEG inputs. This approach allowed for the classification of raw EEG data without requiring hand-engineered features or complicated signal-processing procedures.	Although the study relied on single-channel EEG readings to simplify the procedure, it is possible that this approach only captures part of the complexity of brain activity as multi-channel EEG systems would. This may reduce the accuracy and robustness of sleepiness detection in various scenarios.
[64]	2021	Simulator	9 Subjects		EEG	Vigilance AND drowsiness detection	1D-CNN and 1D-UNet-LSTM	The authors suggested a 1D-CNN-LSTM model that combined the advantages of convolutional neural networks (CNNs) and long short-term memory (LSTM) networks. This model addressed the long-term dependency difficulties inherent in EEG signals, which improved the model's capacity to reliably characterize alertness stages.	The research concentrated on the proposed designs (1D-UNet and 1D-UNet-LSTM) without providing a thorough comparison to a larger variety of existing models. This could limit our understanding of how these models perform relative to other cutting-edge approaches in the field.
[65]	2021	Simulator	Used two pubic data set (SEEDVIG and PSAED)		EEG	Drowsiness	DNN	The research proposed a method for picking a good single channel from numerous EEG channels. This method analyzes different signal properties, such as signal strength, distribution variability, and correlation, to select the optimum channel for drowsiness detection.	The success of the channel selection strategy is heavily reliant on the features retrieved from EEG signals. If the selected features do not accurately represent the underlying brain activity associated with drowsiness, classification performance may suffer.
[66]	2021	Actual driving	16 subjects	1 h	EEG	Drowsiness	CNN	The paper presented a 12-layer deep convolutional neural network (ConvNets) model that automatically learned and extracted features from raw EEG data, eliminating manual feature selection requirements. This end-to-end strategy simplifies the process of detecting tiredness by combining features. extraction and classification into a single model.	The study included young male volunteers, limiting the findings' applicability to other demographics like females or senior drivers. Furthermore, the dataset utilized to train the model is relatively tiny, demanding data augmentation approaches to compensate for the lack of data, which may compromise the model's robustness.
[67]	2021	A visual-reality environment	37 Subjects		EEG	Fatigue	4-D CNN	This study presented a unique 4-D Convolutional Neural Network (4-D CNN) framework for analyzing brain dynamic states and predicting driving performance. This approach recorded both geographical and temporal dynamics of brain activity data, allowing for precise predicting of driving behavior.	The study collects data in a simulated virtual reality environment, which may not fully depict the complexities of real-world driving environments. Furthermore, while the 4-D CNN outperformed other models, it needs significant computer resources, limiting its practical implementation in real-time, in-vehicle systems.
[68]	2021	Actual and Simulated driving	269 Subjects		EEG and EOG	Sleepiness	CNN with LSTM	The study presented a deep neural network model that uses EEG and EOG signals to identify drowsy states accurately. The research also contributed by developing a comprehensive framework for feature extraction and model training, which improved the accuracy and reliability of tiredness detection in drivers.	The training dataset included data from both real-world and simulated situations, which, while valuable in data size, may impact the model's generalizability due to variances between both settings. Furthermore, the study needed to compare its findings to existing sleepiness detection systems fully.
[69]	2019	Simulator	10 subjects		EEG	Fatigue	LightFD based on gradient boosting framework (Light-GBM)	This model integrated a common spatial pattern (CSP) with a gradient-boosting framework to achieve excellent accuracy and efficiency in categorizing EEG signals associated with various mental states, such as alertness and drowsiness. The LightFD model outperforms classic classifiers.	This study collected data in a simulated driving environment, which may not accurately reflect the complexity of real-world driving conditions. Furthermore, while the LightFD model shows promising results in speed and efficiency, it requires further validation on larger datasets and in real-world scenarios.
[70]	2019	Simulator	8 subjects	90 min	EEG	Fatigue	EEG-based spatial–temporal convolutional neural net- work (ESTCNN)	This study developed a spatiotemporal convolutional neural network (ST-CNN) that effectively captures both spatial and temporal patterns from EEG signals, increasing the accuracy of driver fatigue recognition. The proposed model employed a hybrid structure that combines CNN and LSTM layers to extract comprehensive features.	The study's model is tested on a small dataset, which may not represent the full range of driving scenarios and driver actions, limiting its generalizability. Furthermore, the suggested approach necessitates considerable computational resources for training and deployment, which may provide difficulties for real-time vehicle applications.
[71]	2016	Simulator	20 subjects	90 min	EEG	Fatigue	Recurrent self-evolving fuzzy neural network (RSEFNN)	Using EEG data, the study developed a Recurrent Self-Evolving Fuzzy Neural Network (RSEFNN) model to predict driving weariness. The RSEFNN used both spatial and temporal firing layers to capture both static and dynamic patterns of brain activity successfully.	The availability and quality of training data limit the model's effectiveness, especially given the inherent heterogeneity in individual drivers' physiological and cognitive reactions to fatigue.
[72]	2015	Actual driving	16 Subjects	3.5 h	EEG	Fatigue	Pulse coupled neural network	To manage imbalanced data, the study proposed a system that integrated adaptive synthetic sampling and a random forest classifier, assuring stable performance over a range of driver fatigue levels.	The study's validation is mainly based on simulated driving situations, which may replicate only some of the intricacies and variables in real-world driving environments. Individual variances in EEG patterns and the presence of noise or abnormalities in EEG signals may all impact the system's performance.
[73]	2014	Simulator	20 Subjects	2hrs	EEG, EMG and EOG	Fatigue	Complexity Measures and Neural Networks	This study developed a real-time system for identifying driver fatigue by analyzing multiple entropy and complexity measurements applied to EEG, EMG, and EOG signals. The study included new features, including wavelet entropy (WE), peak-to-peak values of approximate entropy (PP-ApEn), and sample entropy (PP-SampEn).	The limitations include the need for high-quality EEG, EMG, and EOG signals, which may not always be possible in real-world conditions due to noise and signal interference. Furthermore, the trials took place in a simulated driving environment, which may not fully duplicate the complexity and variety of real-world driving conditions, limiting the results' generalizability.

**Table 3:** Hybrid measurements.

Ref	Year	Experimental Setup	Number of Subjects	Driving Duration	Type of Sensors	Physical Measurand	Detection Algorithm	Problem Solved	Limitations
[74]	2023	Simulator	13 Subjects	1 h	thermal camera, RGB camera, and environment thermometer	Drowsiness	YOLOv5	This study developed a vigilance detection system for drivers of autonomous rail rapid transit (ART) vehicles that combines face thermal imaging with ambient data. Thermal imaging was utilized to record physiological data, including skin temperature and respiratory patterns.	This work's limitations were its reliance on thermal imaging, which, while helpful in recording physiological signals, can be susceptible to external temperature changes and may require precise calibration and controlled circumstances to get reliable results.
[75]	2022		Used an online public data set and 2 subjects		Camera and AD8232 heart-rate sensor	Fatigue	A customized convolutional neural netWork	The system used the Nvidia Jetson Nano developer kit and Arduino Uno for embedded computing, combining eye and mouth localization techniques with heart rate monitoring to detect tiredness and the presence of a face mask accurately.	Although the system was designed to work in various situations, its effectiveness may be reduced in situations with poor lighting or different viewing angles. The heart rate monitoring module requires accurate electrode placement, which may not always be convenient or comfortable for users.
[76]	2021	Simulator	Public data set (SEEDVIG) and 23 Subjects		EEG and EOG electrodes And SMI eyetracking glasses	Driver's Vigilance and Fatigue detection	A capsule attention mechanism with (LSTM)	A capsule attention method is developed, allowing the model to focus on the most critical aspects of the learned multimodal representations. This multimodal system combines EEG and EOG data for real-time driver vigilance evaluation.	The inclusion of EEG and EOG data complicates the model. The research noted that multimodal analysis is complicated due to including complementary and conflicting information in the signals.
[77]	2020	Simulator	Public data set		Camera and ECG sensors	Fatigue	(CNN) and deep Belief network (DBN)	The authors developed a Hybrid Fatigue system that integrated visual information, such as the PERCLOS measure, with non-visual features, particularly heart-rate signals from ECG sensors.	The suggested approach is extremely dependent on the quality of the sensors employed. The system's performance may suffer if the sensors fail to record reliable data due to environmental conditions or hardware problems.
[78]	2019	Simulator	21 subjects	110 min	Physiological sensors, Camera	Drowsiness	Artificial neural network models	The study utilized a range of data sources, including physiological indicators (heart rate, breathing rate), sensorimotor indicators (blink duration, PERCLOS), and driving performance measurements (lane position, steering wheel angle).	Using a controlled, monotonous driving simulator setting was one of the drawbacks since it may need to fully capture the complexity of real-world driving situations and their impact on drowsiness detection and prediction.
[79]	2018	Simulator	29 Subjects		Infrared camera and PVT	Drowsiness	CNN	The system used convolutional neural networks (CNNs) to extract data-driven features associated with eye closure dynamics across four timescales (5 s, 15 s, 30 s, and 60 s), allowing it to balance accuracy and responsiveness.	The drawbacks included the reliance on video-based facial analysis, which can be affected by differences in lighting conditions, head positions, and potential occlusions, such as glasses or facial hair, influencing the accuracy of eye closure recognition.

**Table 4:** Motion sensors.

Ref	Year	Experimental Setup	Number of Subjects	Driving Duration	Type of Sensors	Physical Measurand	Detection Algorithm	Problem Solved	Limitations
[80]	2024	Actual driving	9 Subjects	Total of 73 trips (0.5–10 h each trip)	wristbands, vehicle mounted equipment, and trip logs	Fatigue	Attention-BiLSTM	The authors developed a fatigue detection technique that incorporates non-visual features from customized wristbands, vehicle-mounted devices, and trip logs. This strategy solves the drawbacks of conventional methods that rely mainly on visual and physiological aspects, which can be obtrusive and less trustworthy.	The method for assessing feature relevance has inherent limitations. Certain qualities may only be beneficial when paired with others, resulting in an underestimate of their individual importance. This could have an impact on our general understanding of which traits are most important for detecting fatigue.
[81]	2022	Simulator	15 Subjects	1 h	Motion Capture Sensor the head pos	Fatigue and drowsiness	reLU-BiLSTMs	The study compared the performance of separate day and night models to a combined model and found that the models performed similarly. This finding implies that separate models for distinct driving conditions (daytime vs. midnight) can be useful for fatigue identification.	One important drawback is that the same dataset was used to construct the sleepiness detection algorithm and evaluate its performance. This dual use can produce biased results that do not accurately reflect the model's performance in real-world circumstances.
[82]	2021	Actual and simulated driving	5 subjects		microphones and speakers of smartphones	Drowsiness	LSTM networks	This work developed a drowsy driving detection system using only the embedded audio components (microphone and speaker) on cellphones. To identify tiredness in real time, the system recognized specific Doppler shift patterns induced by typical drowsy behaviors such as nodding, yawning, and aberrant steering wheel motion.	The reliance on smartphone audio devices to detect Doppler shifts may be susceptible to background noise, changes in driving situations, and smartphone location within the vehicle, potentially impacting detection accuracy.
[83]	2021	Simulator	40 subjects		impulsive radio ultrawideband (IR-UWB) radar	Drowsiness	Different Machine learning and Multilayer Perceptron	The study proposed a non-invasive device for detecting driver drowsiness based on Impulse Radio Ultra-Wideband (IR-UWB) radar technology. This technology recorded chest movements to determine breathing rates, allowing for precise sleepiness detection.	Using Impulse Radio Ultra-Wideband (IR-UWB) radar, required precise location and environmental conditions. Furthermore, major driving movements or body position changes that alter radar signal quality may jeopardize the system's performance.
[84]	2019	Actual driving	8 subjects		Steering wheel angle sensor (SWA)	Fatigue	Fuzzy recurrent neural network model (RNN)	The author developed a fuzzy recurrent neural network (FRNN) model that detects driver weariness using steering-wheel angle (SWA) data. This model captured the nonlinear properties and anomalies in SWA time series data under real-world driving settings.	The limitations of this work include the difficulties associated with relying simply on steering-wheel angle (SWA) data, which can be influenced by different external factors such as road conditions, driver habits, and vehicle dynamics, potentially reducing the accuracy of fatigue detection.

**Fig. 3:**
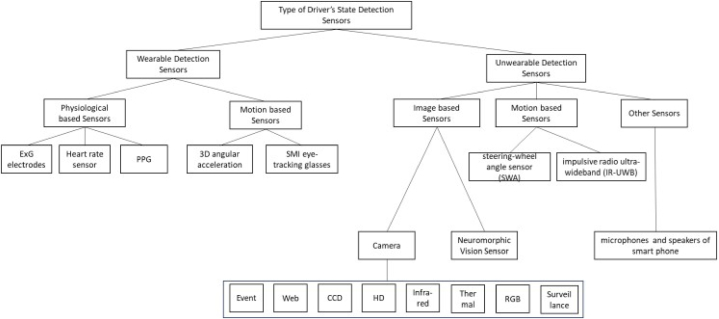
Research outcomes described the types of the sensor's technology implemented in the selected publications.

**Fig. 4:**
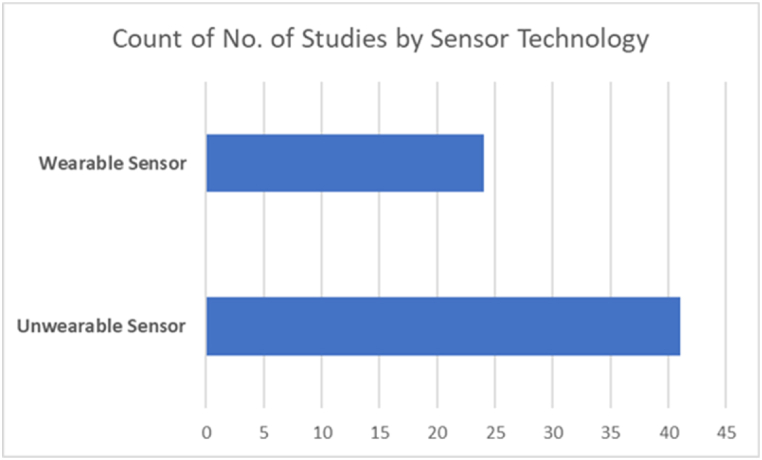
Comparison of wearable and unwearable sensor technology implemented in the selected studies.

Fig. 3 illustrates the research findings and answers the first research question. Therefore, the kinds of sensors implemented in various ways, wearable types based on the detection methodology, and unwearable types represent the two primary components of driver fatigue detection. The results of this study also reveal the superiority of unwearable sensor technology for fatigue and drowsiness detection because of its non-intrusive capabilities for capturing the drivers’ state. Fig. 4 shows the comparison of the wearable against unwearable sensor implementations in the selected studies.

### 3.1 Wearable driving vigilance detection system

**Fig. 5:**
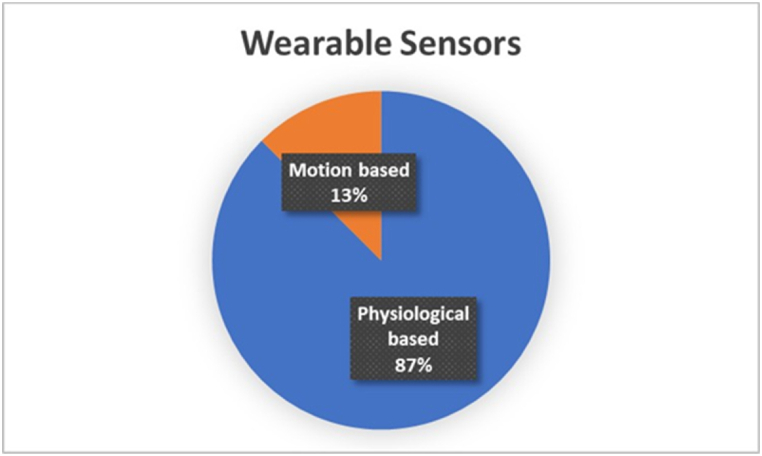
Percentage of wearable sensor’ implemented in the selected studies.

This section demonstrates wearable sensing technology, such as physiological and motion sensors. The characteristics of each technology and its application are discussed. Fig. 5 illustrates the percentage of wearable sensing technology in the selected studies, and it shows that the physiological sensing approach is implemented more than other wearable sensing techniques in the driver's state detection.

#### 3.1.1 Physiological based wearable sensors

Thanks to the biological capacity to perceive physical situations and the progress in sensor technology, physiologically based techniques for capturing physical and mental data from the human body have become widely used in detecting driving fatigue. These approaches make use of a variety of physiological signals. The quantities mentioned are electrooculogram (EOG), electrocardiogram (ECG), and electroencephalogram (EEG). These signals may accurately quantify changes in eye activity, heart rate, and brain activity correlated with fatigue [85]. Electrooculography (EOG) is a bioelectrical signal generated by the movement of the eyes, which can be detected by measuring the electrical activity in the skin surrounding the eyes. The EOG signal possesses abundant data that can accurately indicate the level of driving fatigue [86,87].

**Fig. 6:**
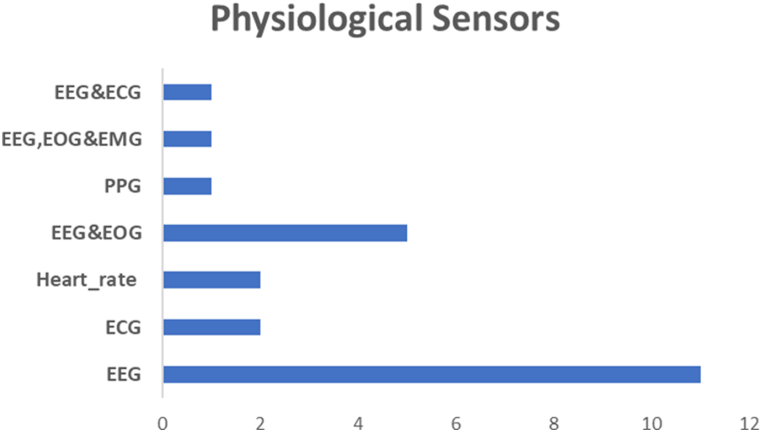
Distribution of the physiological sensors' implementation in the selected studies.

An electrocardiogram (ECG) is a harmless signal that can be obtained without causing any harm to drivers while they are driving. ECG signals are readily obtainable and less intrusive than other physiological signals. Researchers have developed numerous features to distinguish different internal states and pathological diseases of the heart. The Heart Rate Variability (HRV) signals obtained from ECG data exhibit a high level of noise resistance and have been proven to be a reliable indicator for distinguishing internal states. A thorough understanding of heart rate variability is produced using linear and nonlinear analysis approaches in the time and frequency domains (HRV) [88]. Many researchers have focused their efforts on using HRV [89] to identify fatigue, inattention, and drowsiness, [[90], [91], [92], [93], [94]]. Significant physiological information is obtained from the photoplethysmography (PPG) signal, which is influenced by mental state through the autonomic, vascular, and cardiac nerve systems. PPG signals are recorded by pulse oximeters, frequently used in medical settings to track pulse rate and arterial blood oxygen saturation. These popular devices are simple to combine with various technologies, such as tablets, smartphones, and health monitors [95]. PPG sensors assess variations in blood volume with each pulse using a light source and photodetector, whereas ECG sensors measure the electrical signals produced by the heart. PPG offers a clear convenience benefit over ECG since it can monitor impulses at the wrist, fingertip, and earlobe, among other peripheral body locations. On the other hand, ECG requires the uncomfortable placement of electrodes [96] on the other side. Electroencephalography (EEG), a method of recording electrical brain activity through scalp electrodes, can be employed for mental state detection. Electrodes capture EEG signals, which are the fluctuating electric fields generated by the brain [97]. Fig. 6 illustrates the distribution of the various physiological sensors for driver's state detection.

The most psychological signal used in the driver's state in recent research is the EEG signal due to the comprehensive and reliable information that this signal implies. Therefore, to accurately identify driver's fatigue in a real-life driving situation, Wang F et al. [62] proposed a driving fatigue classification algorithm based on the wavelet scattering network (WSN). Initially, EEG data from 12 people were collected in the actual driving environment and divided into two categories: fatigue and exhaustion alertness. Furthermore, the WSN approach extracts wavelet scattering coefficients from EEG signals fed into a support vector machine (SVM) as feature vectors for classification. In another work, Balam V et al. [63] introduced a method for automatically detecting fatigue using single-channel EEG data. Initially, the author analyzed pre-recorded sleep state EEG data acquired from an established dataset. Subsequently, a deep learning framework was employed, utilizing a convolutional neural network (CNN) to carry out the classification phase. The author suggested carrying out validations on a subject-specific basis across different individuals and combining data from multiple subjects to enhance the generalization. The experimental results demonstrate a promising finding for fatigue and drowsiness identification using single-channel EEG signals.

The authors in Ref. [64] presented a technique for forecasting people's levels of mental state using EEG readings and deep learning architectures. Two types of networks were built: a 1D-UNet model consisting solely of deep layers of a 1D convolutional neural network (1D-CNN) and a 1D-UNet-long short-term memory (1D-UNet-LSTM) model that combines the LSTM recurrent model with the proposed 1D-UNet architecture. The experimental findings demonstrate that the proposed models can precisely identify each subject's level of alertness. With 1D-UNet and 1D-UNet-LSTM, each class's average precision and recall can be as high as 86 % and 85 %, respectively. As mentioned before, various research studies have demonstrated the utilization of EEG signals and artificial intelligence algorithms to identify the driver's condition. Although there are numerous hurdles in the experimentation process, decreasing the number of recorder channels for more convenient use in real-time applications is strongly recommended.

Balam V et al. [65] provided a technique for channel selection in a single-channel EEG-BCI system that considers the statistical characteristics of the EEG data from every available channel. A deep neural network (DNN) classifier was also created via the stack ensemble technique to improve classification accuracy. The proposed model was validated with the Simulated-virtual-driving driver and physio net sleep analysis EEG datasets (PSAEDs). They applied a variety of validations, including subject-wise, cross-subject-wise, and combination subject-wise validations, to improve the generalization potential of the presented approaches. Selecting EEG channels can result in a limited dataset, particularly when inputting it into a deep learning model. A data augmentation technique is suggested to address the limited availability of extensive EEG data, as reported in Ref. [66]. To extract essential features from raw EEG data, the author developed a 12-layer deep ConvNets model that consists of 5 convolutional layers, 3 max-pooling layers, and 1 mean pooling layer. The approach also maximizes classification results across three fully connected layers. The deep ConvNets model is trained on 4-s segments of EEG data from multiple persons and tested using 10-fold cross-validation. On the testing data set, the deep model achieved 97.02 ± 0.0177 % accuracy, 96.74 ± 0.0347 % precision, 97.76 ± 0.0168 % sensitivity, 96.22 ± 0.0426 % specificity, and a mean f-measure of 97.19 ± 0.0157 %. Another approach for utilizing multimodal physiological signals such as EEG, EOG and ECG for driving vigilance detection. However, researchers face challenges in implementing such an approach, such as noise and artifacts. EEG and EOG signals are often contaminated by environmental noise and artifacts, including those caused by motion and muscle activities. Additionally, multimodal complexity analysis, such as the difficulty in analyzing EEG and EOG data simultaneously due to the need to identify complementary and contradicting information within these signals.

Moreover, the variability in biological Signals, where EEG and EOG are highly participant-dependent, varies significantly between individuals and even for the same individual at different times. To address the challenges mentioned above, researchers proposed different solutions. For instance, G.i Zhang et al. [76] developed a deep learning model based on a capsule attention mechanism to focus on the most salient features in the multimodal EEG-EOG data, enhancing the robustness against noise and artifacts. To effectively handle the complexity of multimodal analysis and extract temporal features from EEG and EOG signals, the authors utilized a deep Long Short-Term Memory (LSTM) network. Nevertheless, adding Long Short-Term Memory (LSTM) to the Convolutional Neural Network (CNN) introduced greater complexity to the classifier. This approach theoretically has the potential to achieve higher accuracy due to LSTM's ability to account for time dependencies in the signals, but it also necessitates a more significant number of training examples to be effective.

Hultman M et al. [68] performed a comprehensive investigation with many participants and several experiments. They collected data from 12 tests comprising 269 drivers and 1187 driving sessions. The results were collected during the daytime (low sleepiness) and nighttime (high sleepiness) periods. The data was obtained during genuine driving scenarios on actual roads or in a sophisticated driving simulator. The deep neural network was fed with EOG and EEG time series data, divided into 16634 segments of 2.5 min each. The authors also studied the potential of using separate CNN networks for EOG and EEG data modalities. Nonetheless, there was an insignificant improvement in performance when using both EOG and EEG data in channels for the same CNN. This demonstrates the challenge of developing a network structure that can adequately process and combine multiple types of electrophysiological data. Another technique proposed by Song K et al. [98] addresses the difficulty of merging EEG and EOG modalities. The author developed a deep coupling recurrent autoencoder (DCRA) to do this. The coupling layer, which connects EEG and EOG data, is critical for combining these two modalities. This layer is critical for merging data from many sensors. It includes a joint objective loss function necessary for auto-encoder training. This function consists of three components: multi-modal loss (S), EOG loss (LO), and EEG loss (LE). The Mahalanobis Distance was utilized to assess the comparability of the two distinct modal data types. Subsequently, the auto-encoder was constructed using the Gated Recurrent Unit (GRU), which aids in preserving data integrity over the long term.

In contrast, ECG signals reveal heart activity and offer information on the autonomic nervous system's reaction, which is also influenced by fatigue. These measurements offer a more accurate and consistent method to identify fatigue when combined with EEG signals, particularly in high-stress activities like driving, where performance can drastically deteriorate, and the likelihood of accidents increases. To increase the reliability and accuracy of detecting driver fatigue—a crucial factor in maintaining traffic safety and averting collisions G. Du et al. [61] have integrated EEG and ECG data. This resulted from applying the Product Fuzzy Convolutional Network (PFCN), an advanced deep learning framework that processes and analyzes EEG and ECG information to diagnose driver fatigue accurately. The PFCN framework consists of three sub-networks: the first subnetwork processes EEG signals using a fuzzy neural network with feedback and a product layer; the second subnetwork processes ECG signals using 1-D convolution; and the third subnetwork integrates EEG and ECG features while reducing noise interference using a fusion-separation mechanism.

#### 3.1.2 Motion based wearable sensors

Since motion sensors require no direct physical touch with the driver, they are less intrusive than physiological sensors like EEG or ECG. This reduces discomfort and the intrusive nature of the monitoring process, making it more suitable for real-life driving environments. Moreover, for real-time implementation, wearable sensors like those used for motion capture provide fast and accurate information about the driver's state without interfering with their comfort. This enables real-time monitoring of driver behavior. Wearable motion sensors record exact movement data by carefully placing them on crucial regions of the driver's body, typically the head. These sensors track and log a variety of properties, including head position, orientation, and movement velocity.

The literature has examined innovative ways for real-time driver behavior monitoring utilizing wearable sensors. For example, it includes a bracelet that monitors skin conductance and body temperature [99], a Google Glass-based gadget that estimates eye blink frequency [100], and intelligent headgear with RFID sensors that detect head rotation and nodding [101]. Motion sensors were used to track the human body's motion, including head position, eye, hand, and lower limb movements, to identify mental fatigue. S. Ansari et al. [81] presented an MCS for monitoring drivers' head posture changes and motions as signs of mental tiredness. This system contains wearing sensors on the driver's head, forearms, shoulders, and right foot. A driver-in-loop (DIL) simulator interfaced with Unreal Engine provided a regulated yet realistic driving environment for data gathering from 15 healthy volunteers. Based on a rectified linear unit(reLU) layer, a novel modified bidirectional long short-term memory (BiLSTM) deep neural network analyzes the acquired data, namely 3D time-series head angular acceleration data. Concerning training accuracy (99.2 %), sensitivity (97.54 %), precision (97.38 %), and F1 scores (97.46 %), the suggested network performed admirably.

On the other hand, the SensoMotoric Instruments eye-tracking glasses (SMI-ETG) are a significant factor in eye movement tracking. Utilizing the SMI eye-tracking glasses, one may compute the PERCLOS index, a commonly recognized measure of attentiveness. The percentage of eye closure over time, or PERCLOS, is a trustworthy measure of how alert and tired a driver is. The SMI offers comprehensive data on several kinds of eye movements. The PERCLOS formula combines the measures of blinks, saccades (fast eye movements), fixations (constantly focused eyes), and CLOS (closed eye duration) to provide a complete picture of the driver's level of awareness. Wu W. et al.’s [102] analysis used Forehead Electrooculogram (EOGF) signals obtained using forehead electrodes. Because this technique does not require electrodes around the eyes, it is less intrusive and more practical. The authors introduced a subnetwork node-augmented deep autoencoder model. This network primarily focuses on dimension reduction and sparse representation. The proposed approach utilizes SMI eye-tracking glasses, which can record up to 120 frames per second, to monitor real-time changes in eye closure percentage. By utilizing three distinct EOGF characteristics, the study demonstrated a significant enhancement in the correlation coefficient (COR) and root-mean-square error (RMSE).

### 3.2 Unwearable driving vigilance detection system

**Fig. 7:**
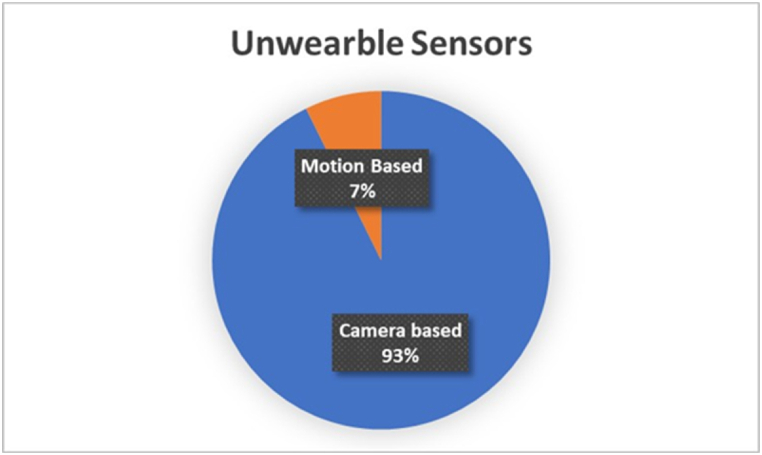
Distribution of the physiological sensors' implementation in the selected studies.

The Unwearable Driving Vigilance Detection System significantly advances driver attention monitoring, differentiating itself from wearable sensor technology. Unwearable systems operate remotely and do not require drivers to wear accessories like caps, wrist-bands, or glasses. This distinction increases driver comfort and convenience while ensuring continuous monitoring without relying on user compliance or the possibility of forgetting to wear the device. Installing cutting-edge cameras and sensors inside the vehicle and placing them strategically to capture a complete picture of the driver's face and upper body is necessary for implementing unwearable systems. These systems use sophisticated computer vision and artificial intelligence algorithms to identify fatigue indicators by evaluating visual data. Unwearable technology's non-intrusive design guarantees a smooth and unnoticeable detecting process, preserving the genuine driving experience while boosting safety. By minimizing wearable sensor downsides like pain and frequent maintenance and charging, this method offers a workable and efficient alternative to increase traffic safety. Fig. 7 illustrates the percentage of unwearable sensors implemented in the selected papers.

#### 3.2.1 Computer vision based (image- or video-based analysis)

One significant advancement in vehicle safety is using image- or video-based driver status detection systems, driven by the urgent need to reduce accidents caused by fatigued or drowsy drivers. These systems continuously monitor drivers using sophisticated cameras and image-processing algorithms. It is essential to use photos or videos since they offer instant visual insight into the driver's actions and physical state. By detecting tiny changes in the driver's head movements, eye behavior, and facial expressions, this technology assists in promptly diagnosing medical issues such as abrupt incapacitation, fatigue, and lack of attention. Progress in computer vision and deep learning enhances the reliability of these systems by enabling accurate processing of complex visual data. Unlike traditional methods that rely on sensors attached to the vehicle or the driver, image- or video-based systems offer a discreet yet efficient surveillance approach. They can function in various lighting and driving conditions, enhancing their versatility and effectiveness.

**Fig. 8:**
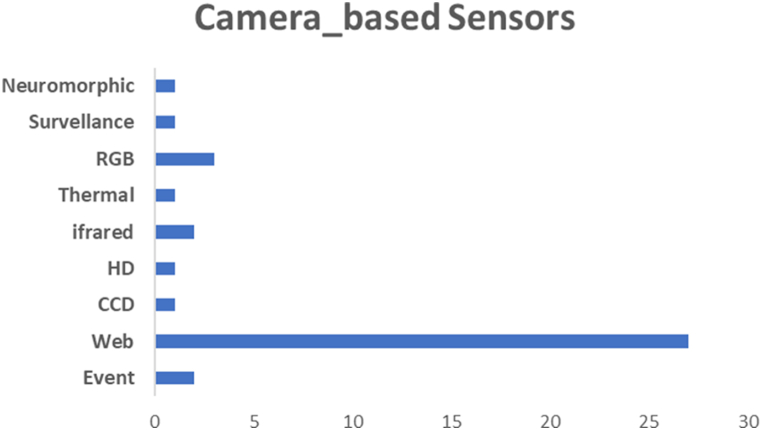
Various camera types that implemented in the selected studies.

Implementing image- or video-based driver state detection systems is a proactive method to enhance road safety, reduce accidents, and improve the driving experience. Various cameras have been implemented in previous studies to capture the driver's states, as depicted in Fig. 8.

Various methods were used in the camera-based technique to detect the driver's states. These methods can be categorized according to the objects that need to be tracked, such as facial expressions and head movements. Eye movement in unique patterns can indicate fatigue or drowsiness. M. Q. et al. [79] used facial expressions, particularly eye closures, to detect drowsiness. The authors used off-the-shelf algorithms to extract an eye image for each eye frame. It involves detecting the face region, localizing facial landmarks, computing the eye positions, and extracting the eye images using affine warping. The final output is an eye image of 24 × 24 pixels for each eye. Then, the spatial Convolutional Neural Network (CNN)takes the grayscale eye image as input and outputs an estimate of the eyelid's distance in pixels.

Using specialized modules for eye image processing, eye-lid distance estimation, and a temporal CNN for drowsiness detection improved performance metrics. Focusing on eye closure and head posture, Teyeb I. et al. [56] developed a multi-modal system that combines two visual parameters: eye closure duration and head movement angle to detect the driver's hypervigilance. Using the Viola and Jones method, the system used video data to identify and track the head and eyes, two critical locations of interest. The system consists of two subsystems: a head posture subsystem and an eye blinking analysis subsystem. Together, these elements determine the degree of attentiveness based on the angle of head movement and the moment of eye closure. This technique provides a novel and efficient method for identifying driver hypo-vigilance. The authors used Convolutional Neural Networks (CNN) for feature extraction, The Wavelet Network (WN) for classification, and fuzzy logic for calculating vigilance levels. To address facial expressions, Gue J. et al. [103] presented a method that integrated Long Short-Term Memory (LSTM) networks and Convolutional Neural Networks (CNN). Real-time processing and facial fluctuation resistance were two issues that our hybrid approach successfully addressed. The system consists of a temporal component that tracks these features across time and a spatial component that extracts face elements like the lips and eyes from a single image. By scaling the frame image, using the P-Net architecture, and clipping essential portions of the mouth and eyes to create small images for feature extraction, the suggested system uses Time Skip Combination (TSC-LSTM) to detect faces and identify facial landmarks. The authors noted that the suggested method, particularly the TSC-LSTM with refinement, achieved an impressive accuracy rate of 84.85 %. Furthermore, the instability of visual analysis under varied lighting circumstances is a barrier that must be overcome.

Gue J et al. [103] suggested a condition-adaptive representation learning system to address this problem. The system is based on a 3D deep convolutional neural network (3D-DCNN) and includes four models: spatio-temporal representation learning, scene condition understanding, feature fusion, and drowsiness detection. The technology utilized visual sensors, such as RGB or active infrared sensors, to detect the driver's facial expressions and motions. These sensors are deployed in various locations, including the vehicle dashboard, sun visor, and overhead console, to capture driver facial photos successfully. Subsequently, a 3D-DCNN is employed to extract spatiotemporal representations from video clips, considering both motion and appearance features. Additionally, Deng W. et al. [55] proposed a method to handle lighting conditions or situations where drivers have glasses. The authors introduced” DriCare,” a technology that utilizes facial feature analysis to detect signs of driver drowsiness in real-time promptly. Face tracking was carried out using multiple Convolutional Neural Networks-Kernelized Correlation Filters (MC-KCF). This method combines CNN and KCF to increase tracking accuracy under challenging situations, such as low-light circumstances or when the face moves out of the camera's view. The multitask convolutional neural networks (MTCNN) improve the MC-KCF method to enable robust face tracking. The system achieved an average accuracy of around 92 % in various conditions. The processing speed changed depending on the lighting conditions: 18 fps in bright areas and 16 fps in dark environments.

#### 3.2.2 Motion based sensors

As illustrated in the previous section, sensor techniques used for driver fatigue detection can be broadly divided into wearable and unwearable schemes. Wearable devices assess physiological features such as EEG, EOG, and ECG readings, which can annoy or distract drivers. On the other hand, unwearable systems detect weariness by extracting face features and operating behavior data from devices such as webcams. Unwearable motion sensors provide a non-invasive approach for monitoring drivers’ state. This system is crucial because it eliminates the need for the driver to wear any additional equipment, which could be annoying or distracting. Since they do not demand any action from the driver, such as putting on a device, they are more user-friendly, especially for long drives. Compared to physiological or facial capture sensors, unwearable motion sensors can be fitted in any car without requiring special modifications for various drivers. This detection technique makes them appropriate for shared or commercial vehicles, such as trucks or buses, where driver fatigue is a significant concern.

Furthermore, rather than acquiring physiological data, unwearable motion sensors focus on movement and behavioral patterns, which may attract privacy-conscious users more. One such technology is the driver's steering wheel angle (SWA) methodology for fatigue assessment. Studies have indicated that extended driving might cause a driver's energy levels to drop and weariness to set in. During this period, his capacity to operate or direct the vehicle will be significantly diminished, decreasing the precision and frequency of steering wheel rotation [104,105]. Thus, by acquiring real-time operational data on the driver's steering wheel, fatigue patterns can be extracted from the changes in the direction angle data. Subsequently, building a model for determining the driver's fatigue level allows for effective detection of the driver's fatigue level [106,107]. Using simulator data, Berglund J [108]. conducted fatigue driving studies on 22 participants. The data acquired included state variables from 10 vehicles: SWA (steering wheel angle), steering wheel angular velocity, steering wheel torque, yaw rate, and vehicle lateral position. By utilizing a linear regression model and analyzing 17 specific symptoms of weariness, the accuracy of detecting fatigue reached an impressive 87 %.

However, most existing research, especially those based on steering wheel angle (SWA) data, often uses data from driving simulators, which may not accurately replicate real-world driving conditions. Moreover, these methods primarily focus on statistical features of operation time series, which may not account for individual differences in driving behavior. This lack of robustness in actual vehicle conditions and the challenge of extracting meaningful data from SWA under actual driving conditions were identified as significant gaps. To address this issue, Li Z. et al. [84] focused on developing a robust method for real-time detection of a driver's fatigue state using SWA sensors under actual driving conditions.

SWA data was collected under natural road conditions using the steering-wheel angle sensor, which was then used to analyze the fatigue characteristics of the driver. The authors then constructed a four-layer fuzzy recurrent neural network model to extract deep and meaningful features from the SWA data, considering the individual differences and randomness in actual driving conditions. As a result, the model achieved an average recognition rate of 87.30 % in the fatigue sample database under actual vehicle conditions, indicating strong robustness and effectiveness in different subjects and real driving scenarios.

On the other hand, the performance of vehicle dynamics-based systems is hindered by unpredictable factors like road geometry and traffic. Therefore, researchers have proposed another approach based on unwearable and camera-free methods to detect driver fatigue using unwearable. Siddiqui et al. [83] used an Impulse Radio Ultra-Wideband (IR-UWB) radar system to acquire chest movement data, estimating the respiration rate. This approach aimed to provide a comfortable, distraction-free, and accurate means of detecting driver drowsiness in natural environments. The data comprising RPM, age, and labels (drowsy/non-drowsy) were structured into a dataset. The RPM derived from the UWB radar-acquired chest movement was validated using a commercially available pulse oximeter device. Then, different machine-learning models were trained on the dataset. The best accuracy (87 %) in classifying drowsy and non-drowsy states was reported based on the respiration rate, away from specialized sensors that often require additional, often expensive, hardware like infrared sensors, cameras, or EEG devices. Xie et al. [82] proposed a system, D3-Guard, which utilizes the acoustic sensing capabilities of standard smartphones. The method involved emitting an audio signal from the smartphone's speaker, which then reflected off the driver and was captured by the phone's microphone. The system analyzed the Doppler shift in these signals, which varied with drowsy driving behaviors like nodding, yawning, and specific steering wheel operations. The authors used the Fast Fourier Transform (FFT) for feature extraction from the audio signals. They employed Long Short-Term Memory (LSTM) networks to analyze these features and detect drowsy driving behaviors.

### 3.3 Deep learning base fatigue detection

**Fig. 9:**
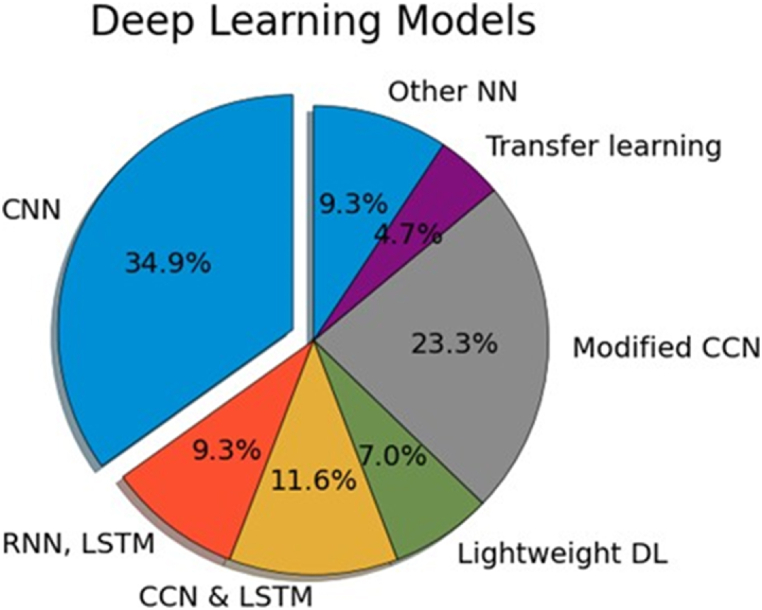
Percentage of deep learning models implemented in the selected studies.

Deep learning (DL) has emerged as a revolutionary tool in enhancing driver vigilance detection, marking a significant stride in machine learning and automotive safety [103]. The motivation for incorporating DL in this domain primarily stems from its exceptional capability to process and analyze vast amounts of complex, unstructured data, a common characteristic of real-world driving scenarios. Traditional machine learning methods often require extensive feature engineering to handle such data. However, DL models, particularly convolutional neural networks (CNNs) and recurrent neural networks (RNNs) excel in automatically extracting and learning features from raw data, be it visual, auditory, or sensory [109]. One of the main advantages of deep learning in driver vigilance detection is its capacity to comprehend visual cues from camera feeds, such as the driver's eye movements [42], head posture, and facial expressions. These indications are critical in determining a driver's attentiveness level. Unlike traditional methods, DL can interpret these signs holistically and integrated, detecting minor changes that may indicate exhaustion or preoccupation. Furthermore, RNNs are adept at interpreting time-series data, including steering patterns and braking behaviors, offering a dynamic knowledge of the driver's attention over time. Furthermore, continuing developments in DL, driven by growing computer power and novel neural network structures, are improving the accuracy and efficiency of these systems. As a result, DL-based vigilance detection systems not only beat their traditional counterparts in terms of accuracy but also provide higher scalability and the potential for integration with other advanced driver assistance systems (ADAS). Various DL models were developed in the selected studies, as illustrated in Fig. 9.

It can be noticed from the figure that most of the applied DL algorithms are CCN or its modified versions, such as Product Fuzzy Convolutional Network (PFCNN) [61], 3D-DCNN [54], or lightweight CNN [29].

### 3.4 Edge computing for driving monitoring

Edge computing plays a significant role in driver's vigilance detection by enabling the real-time processing and analysis of the acquired data immediately within the vehicle or close to it. This approach includes sensors and cameras for the driver's behavior monitoring, eye movement tracking, facial expression detection, and measurement of other physiological signals to evaluate their awareness and attention. A significant improvement in safety driving technology is the integration of edge computing into driver state monitoring systems, which permits a proactive technique to minimize accidents caused by driver fatigue or distraction. Eliminating the need to send large amounts of data to distant machines for analysis increases the safety and responsiveness of the vigilance detection system while also lowering latency and bandwidth usage. Additionally, edge computing techniques make the vigilance detection system safer and more responsive when there is no need to transmit massive amounts of data to remote machines for processing, reducing latency and bandwidth consumption. A. Khan et al. [31] presented a comprehensive, non-invasive Internet of Things-based automated solution for public transportation and logistics use. With its automated fatigue detection, remote monitoring, and driver evaluation features, this system provides a complete solution.

Four primary components comprise the system's architecture and implementation: an embedded system, edge computing, cloud computing, and a user interface. The system identified four driving behavior states with 96 % accuracy: active, eyes closed, yawning, and inattentive. Precision, recall, F1-score, and accuracy were used to evaluate performance in each session. The authors evaluated throughput and latency performance using two embedded boards: the Nvidia Jetson Nano and the Raspberry Pi 4. The study's conclusions demonstrated the best method for detecting and monitoring driver fatigue utilizing computer vision and Internet of Things infrastructure. On the other hand, D. Nguyen [29] used a lightweight CNN to identify the driver's facial and eye areas. The proposed approach began by detecting the driver's face with a real-time face detector known as nano YOLO5Face and then focused on the ocular regions. It used a compact CNN architecture, an inception network, and a triplet attention mechanism for accurate eye detection. The system was validated on an Intel Core I7-4770 CPU and a 128-core Nvidia Maxwell GPU (Jetson Nano device), performing real-time processing speeds of 33.12 frames per second (FPS) and 25.11 FPS, respectively. The authors also provided datasets for the eye detection task, which include 10,659 images and 21,318 labels. The latency and throughput performance measurements were tested using a CPU with VGA resolution and a Jetson Nano device with Full HD (FHD) resolution. The outcome revealed the system's efficiency in real-time processing.

## 4 Discussion

In our comprehensive review of 65 papers on driver drowsiness and fatigue detection, we examined the methodologies based on sensor implementation, detection techniques, real-time capabilities, obstacles, and future directions for vigilance detection systems. Over time, these systems have progressed dramatically, utilizing a variety of sensors, algorithms, and machine-learning models to improve accuracy and reliability. The most frequent procedures involve tracking physiological and behavioral markers such as eye and mouth movements, brain activity (EEG), heart rate variability, head posture, steering behavior, and lane deviation. Each strategy has distinct advantages and disadvantages that vary based on the context of use, data quality, detection algorithms, and evaluation criteria. According to our review results, most of the suggested research collected data in simulated driving environments, with only a few using actual driving environments. Despite the benefits that simulators currently provide, this process for data production lacks real-world elements.

Because traffic accidents are occurrences generated by a set of conditions that are not always the same and occur in location, time, and under specific conditions, the authors may prefer less controlled situations to those offered by simulators to generate data. Data that changes over time, such as traffic accidents, weather conditions, or traffic flow, could be utilized to modify the models’ parameters. For instance, Among the research examined, a few had exceptionally high accuracy rates. Notably, a Neuromorphic vision system based on event camera technology achieved 100 % accuracy in detecting driver drowsiness, demonstrating the promise of new sensor technologies in improving detection performance [35]. Similarly, another study found that integrating a Fotric615C thermal camera with environmental sensors to monitor temperature swings resulted in 99.57 % accuracy [74], while a combination of EEG and EOG sensors similarly achieved 99.3 % detection accuracy. However, while these findings are encouraging, most of this research was verified exclusively in simulated or controlled conditions, raising concerns about their real-world relevance. Controlled environments frequently lack the unpredictable components and diverse situations of real-world driving, such as changing weather, various road kinds, and a wide range of driver behaviors. As a result, the resilience and reliability of these approaches in practical, everyday situations remain unknown.

Moreover, these systems' hardware requirements and potential intrusiveness must be carefully considered. Systems relying on multiple sensors, such as EEG, EOG, and thermal cameras, may incur higher costs and require more complex installation and calibration processes. This complexity could deter widespread adoption due to concerns about user acceptance, increased system costs, and potential impacts on driver comfort and attention. The limitations of the reviewed studies and future directions are described in the following subsections.

### 4.1 Limitations of the existing techniques

Research on driving safety technology identifies several critical barriers to smooth integration and widespread deployment. There is a significant challenge in that the proposed approaches need to be more generalized, limiting their effectiveness in various driving situations and environments. These models are intelligent but could be more accurate at adjusting to the actual world's complex and unpredictable driving situations, which limits their applicability. Additionally, the complexity of the models, computational complexity, and processing requirements prevent real-time application of these technologies, creating a significant obstacle to their adoption. The reliability of the systems is further compromised by their vulnerability to external influences such as noise, lighting, and camera angle. It is common for these systems, which are usually dependent on exact conditions, to break when small changes are made, leaving their robustness and reliability in doubt. Furthermore, physiological data integration for driver monitoring adds another level of complexity as these signals differ significantly from individual to individual and can-not always predict a driver's status due to external circumstances or biological variations. Moreover, practical concerns arise from the requirement for continuous and consistent input quality, which is challenging to maintain in today's dynamic and often unpredictable driving environment. Additionally, the physical condition of the driver, the interior layout of the car, and outside environmental influences are only a few of the variables that have a significant impact on the system's performance and can vary greatly, reducing the accuracy and dependability of the system.

Embedded systems and edge computing are critical components of the driver fatigue detection system, allowing for real-time processing and analysis of driving behavior. Notably, the Nvidia Jetson Nano enables the rapid execution of deep learning models that categorize fatigue states based on facial cues such as mouth and eyes, all within the car. This design not only improves fatigue detection accuracy but also ensures that the system is affordable and practical for general usage in transportation. Furthermore, providing drivers with rapid notifications will significantly improve road safety by lowering the probability of drowsiness-related accidents [33]. However, implementing a driver fatigue monitoring system on such embedded devices has presented some performance issues. For example, on the Nvidia Jetson Nano, the system runs at an average speed of 6 frames per second (fps), much slower than a standard computer's 22 fps. This reduced performance may influence the real-time monitoring abilities required for effective fatigue identification. Furthermore, implementing deep learning models for real-time analysis on an embedded device such as the Jetson Nano requires significant CPU resources. Maintaining system responsiveness requires compelling image processing and model inference within the restrictions of the device's hardware capabilities.

### 4.2 Future directions

A holistic approach should be employed to resolve the constraints, prioritizing im-improving the adaptability, efficiency, and usability of driving safety systems. Future research initiatives emphasize the importance of enriching training datasets, which is critical for enhancing models’ capacity to generalize and perform accurately over a broader range of driving scenarios. Adding a broader range of data that matches the vast complexity of real-world driving can make these models more resilient and versatile. Additionally, long-term system longevity and dependability studies are essential for ensuring constant performance. Thus, these assessments will assist in identifying potential decreases in system performance over time, paving the path for adjustments that ensure long-term reliability and accuracy. Moreover, automating computational models for efficient real-time applications is critical to ensure that these systems operate smoothly and respond quickly when required. Furthermore, simplifying algorithms while maintaining efficiency may result in shorter processing times, allowing these technologies to provide real-time help and avoid accidents more effectively. On the other hand, incorporating non-intrusive and ergonomic sensor designs is crucial for improving user acceptance and comfort. Furthermore, these advancements should be built with a strong emphasis on retaining accuracy and dependability while remaining non-invasive and effortlessly blending into the driving environment. One of the Recommendations is the implementation of multi-modal vigilance estimation methods in real-world scenarios. However, it is essential to ensure they are more robust and interpretable. Additionally, for physiological wearable sensors, it is recommended to utilize a few electrodes to reduce the burden on the drivers and develop a lightweight and low-cost detection system.

Furthermore, they are implementing feedback so that the system can improve on drivers' responses. This mechanism can be introduced by investigating edge computing to conduct real-time data analysis on the vehicle. As a result, this will reduce the reliance on the cloud infrastructure and enhance the responsiveness of the detection system. On the other hand, different suggestions might be employed to address the challenges of the embedded technology utilized to detect driver fatigue. First, adding more sensors, such as accelerometers and heart rate monitors, can provide a more comprehensive assessment of driver fatigue, increasing accuracy. Second, refining deep learning models to develop lighter versions with fewer computational resources would improve performance on devices like the Nvidia Jetson Nano. Third, experimenting with different datasets and lighting circumstances helps improve the system's performance in real-world scenarios, ensuring accurate fatigue detection. Fourth, increasing image processing algorithms for assessing the driver's face, eyes, and mouth can result in higher detection rates, even under challenging settings. Finally, building solutions that are not only functional but also respect the user's privacy and resource efficiency will be critical to the future development and adoption of driving safety technology.

## CRediT authorship contribution statement

**Maged S. AL-Quraishi:** Writing – review & editing, Writing – original draft, Methodology, Investigation, Formal analysis, Data curation, Conceptualization. **Syed Saad Azhar Ali:** Writing – review & editing, Supervision, Methodology, Data curation, Conceptualization. **Muhammad AL-Qurishi:** Writing – original draft, Methodology, Data curation, Conceptualization. **Tong Boon Tang:** Writing – review & editing, Conceptualization. **Sami Elferik:** Writing – review & editing, Conceptualization.

## Data availability statement

No Data associated with this work.

## Declaration of competing interest

The authors declare no conflict of interest.
